# Polymorphisms in Ion Transport Genes Are Associated with Eggshell Mechanical Property

**DOI:** 10.1371/journal.pone.0130160

**Published:** 2015-06-24

**Authors:** Zhongyi Duan, Sirui Chen, Congjiao Sun, Fengying Shi, Guiqin Wu, Aiqiao Liu, Guiyun Xu, Ning Yang

**Affiliations:** 1 National Engineering Laboratory for Animal Breeding and MOA Key Laboratory of Animal Genetics and Breeding, College of Animal Science and Technology, China Agricultural University, Beijing, China; 2 Beijing Huadu Yukou Poultry Industry Co., Ltd., Beijing, China; Temasek Life Sciences Laboratory, SINGAPORE

## Abstract

Eggshell mechanical property traits such as eggshell breaking strength (ESS), eggshell thickness (EST) and eggshell weight (ESW) are most common and important indexes to evaluate eggshell quality in poultry industry. Uterine ion transporters involve in eggshell formation and might be associated with eggshell mechanical property traits. In this study, 99 SNPs in 15 ion transport genes were selected to genotype 976 pedigreed hens of Rhode Island Red. ESS, EST and ESW were measured for each bird at 55 weeks of age. The association study showed that 14 SNPs in 8 genes were significantly related (*p* < 0.05) with at least one trait, and their contributions to phenotypic variance ranged from 0.23% to 4.14%. Both *ATP2A3* and *SLC4A5* had a significant effect on all the three traits. Strong linkage disequilibrium (LD) was detected among SNPs in four genes: *ATP2A3*, *ITPR1*, *SLC8A3*, *SCNN1a*. The significant effects of those diplotypes on eggshell mechanical property traits were found, and their contributions to phenotypic variance ranged from 0.50% to 0.70%. It was concluded that the identified SNPs and diplotypes in this study were potential markers influencing the eggshell mechanical properties, which could contribute to the genetic improvement of eggshell quality.

## Introduction

Eggshell formation and quality has attracted much attention for several decades because of its ubiquity as a biomeralization model [1–2] and significance for poultry industry in reducing the waste due to shell cracking during production and transport [3]. Eggshell breaking strength (ESS), eggshell thickness (EST) and eggshell weight (ESW) are the most direct indexes to evaluate eggshell mechanical properties. The bird eggshell itself is composed of calcium carbonate in calcite form, and has ordered crystal structure, which determines the mechanical properties of eggshell [4–6]. 95% minerals, 3.3%-3.5% organic matrix and 1.6% water constitute the whole eggshell in normal state [7]. The formation of eggshell is initiated by the egg about to migrate into uterus and lasts about 20 h. During this process, amounts of calcium and bicarbonate ions, and precursors of organic matrix are secreted into uterine fluid, where the organic and mineral phases interact and form the eggshell [8–10]. Thus, the biological research on eggshell is divided into two aspects: the organic matrix and the ion transporters in bird uterus.

Recently, several proteomics studies in eggshell and uterine fluid have revealed a mass of eggshell matrix proteins, most of which were considered as either the part of eggshell structure or a regulator for eggshell mineralization [11–14]. Ions transport in respect to eggshell mineralization is studied mainly around the base elements of eggshell: carbonate and calcium [15–17]. The experiment of selective inhibition to ions transfer *in vitro* and *in vivo* [18–19] and the comparisons of uterine ions concentrations among the stages of shell formation [20] suggested that the ions transport in relation to eggshell calcification was a collaboration process of multiple ions transport and regulation, such as Ca^2+^, HCO_3_^-^, Na^+^, and K^+^. A detailed function analysis of ion transport genes by Jonchère et al. (2012) improved the avian uterine ion transport model [2]. According to this model, the function of ion transport genes can be summarized as: (1) Ca^2+^ is transferred by TRPV6 Ca^2+^ channel from blood plasma to uterine glandular cells, then extruded by membrane’s Ca^2+^ pumps (ATP2B1, 2) and Ca^2+^/Na^+^ exchangers (SLC8A1, 3). The endoplasmic Ca^2+^ pumps type 2, 3 (ATP2A2, 3), inositol trisphosphate receptors type 1, 2, 3 (ITPR1, 2, 3) and 28 kDa calbindin (CALB1) maintain a low intracellular free Ca^2+^ concentration. (2) Three Na^+^ channels (subunits SCNN1a, 1b, 1g) and Na^+^/Ca^2+^ exchangers SLC8A1, 3 involve Na^+^ uptake in the cell, and the Na^+^/K^+^ ATPase (ATP1A1, ATP1B1) output Na^+^ from the cell. (3) The Na^+^/K^+^ ATPase take K^+^ in the cell. The K^+^ channels (KCNJ2, 15, 16 and KCNMA1) excrete K^+^ at the apical membrane. (4) CA2 involves the production of HCO_3_^-^ from CO_2_. The Na^+^/HCO_3_^-^ co-transporters (SLC4A4, 5, 10) and the HCO_3_^-^/Cl^-^ exchanger SLC26A9 contribute to the transport of HCO_3_^-^. H^+^ produced during the reaction of HCO_3_^-^ are transferred to plasma via the membrane Ca^2+^ pumps ATP2B1, 2 in the apical membrane and the VAT pump at the basolateral level. (5) The HCO_3_^-^/Cl^-^ exchanger SLC26A9 transfers Cl^-^ into the cell, and Cl^-^/ H^+^ exchanger (CLCN5) extrudes it.

The microarray study on gene expression profiling in hen’s uterus during eggshell formation revealed more candidate ion transport genes, which were considered probably associated with eggshell mineralization [21]. However, the relation between those candidate ion transport genes and eggshell mechanical properties is not very clear. Based on previous researches on uterine ions transport [2, 21], we selected 15 genes which have been confirmed with significant different expression in uterus compared with magnum and duodenum to detect the phenotypic-genotypic association in a pedigreed line of laying chickens. The aim of this study was to provide evidence for the important role of candidate ion transport genes on eggshell mechanical properties.

## Materials and Methods

### Ethics Statements

All the blood samples were collected from brachial veins of chickens by standard venipuncture. The whole procedure was performed according to regulations and guidelines established by the Animal Care and Use Committee of China Agricultural University. The entire study was approved by Animal Care and Use Committee of China Agricultural University (permit number: SYXK 2007–0023).

### Birds and Phenotypes

Nine hundred and seventy-six hens from the 9^th^ generation of a pedigreed line of Rhode Island Red were used from Beijing Huadu Yukou Poultry Breeding Co. Ltd., China. Feed and water were provided *ad libitum* for all birds during the entire experimental period. All hens were randomly allocated to individual cages. At 55 weeks of age, a total of 2810 eggs were collected for 4 consecutive days from the experimental population to make sure at least 2 eggs for each hen. ESS (kg/cm^2^) was determined using the eggshell force gauge (model-II Robotmation Co. Ltd., Tokyo, Japan) with the blunt end up. ESW (g) was measured with membrane on it and the egg white removed. EST (mm) was measured at three points of eggshell (the equator, sharp and blunt ends of egg) without the membrane.

### Genes and SNPs Selection

Fifteen candidate genes which had a significant differential expression level in uterus compared with magnum and duodenum were selected from the results of Jonchère *et al*. [2, 21]. Detail information of SNPs in those fifteen protein-coding genes was obtained from UCSC Genome Browser database (http://genome.ucsc.edu/cgi-bin/hgGateway). Variants on genes can be categorized as follows: intergenic, upstream/downstream of gene, 5’ or 3’ UTR (untranslated region), CDS (coding sequence)-synonymous coding change, CDS-non-synonymous, intron, splice site or splice region, exon of non-coding gene (http://genome.ucsc.edu/cgi-bin/hgVai). Except for the known function-change mutations, SNPs in CDS and UTR regions are more likely to affect function of genes. We selected ninety-nine SNPs localized in UTR regions and exons of these 15 genes for further association analysis (S1 Table).

### Genotyping and Quality Control

Genomic DNA was extracted from the blood samples using a standard phenol-chloroform method, then quantified using a NanoDrop spectrophotometer (GE Healthcare Life Sciences, Uppsala, Sweden), and the final concentrations were 30~50 ng/μL. Primers were designed using software Assay Design 3.1 (SEQUENOM, San Diego, CA) for each SNP, as shown in S1 Table.

Genotyping of the 976 hens was performed using matrix-assisted laser desorption-ionization time-of-flight mass spectrometry on the Mass ARRAY iPLEX Plat-form (Sequenom, San Diego, CA). Single nucleotide polymorphism with a genotype call rate < 90% and minor allele frequency < 1% across all individuals were removed.

### Statistical Analysis

#### LD Analysis and Haplotype Reconstruction

The linkage disequilibrium (LD) among SNPs within each gene was determined by using Haploview program [22] and assessed by the Lewontin D' statistic and squared correlation statistic r^2^. D' > 0.8 and r^2^> 1/3 means strong LD [23]. The haplotypes for SNPs in strong LD were inferred using Phase (v2.1.1, **http://stephenslab.uchicago.edu/software.html**) [24–25].

#### Association Analysis

The association of single SNP and diplotype with eggshell mechanical property traits were performed with the least-squares method using a linear mixed model (Mixed procedure, SAS version 9.2, SAS Institute Inc., Cary, NC). The linear mixed model was follow:

$$y_{ij}=\mu +h_{i}+g_{j}+a+e_{ij}$$

where *y*_*ij*_ represents the observed values of the traits, μ the population mean, h_*i*_ the fixed effects of house, g_*j*_ the fixed effects of genotype (for the association analysis of SNP with eggshell traits) or diplotype (for the association analysis of diplotype with eggshell traits), a the residual polygenic effects and e_*ij*_ the residuals. The additive genetic relationship matrix was calculated from the pedigree.

#### Variance Components Estimation

The variance components were estimated using the model below and the SAS 9.2 with the restricted maximum likelihood method for the SNPs or diplotypes showing statistically significant association with eggshell mechanical property traits. Then, SNPs or diplotypes contributions to phenotypic variance (CPV) were calculated by the equation CPV = V_g_/V_p_, where V_g_ and V_p_ were the SNP or diplotype and phenotypic variance respectively.

$$y_{ij}=\mu +h_{i}+g_{j}+e_{ij}$$

where *y*_*ij*_ represents the observed values of the traits, μ the population mean, h_*i*_ the fixed effect of house, g_*j*_ the fixed effect of genotype or diplotype, and e_*ij*_ the residuals.

## Results

### Phenotypic Analysis

Descriptive statistical analyses for the three traits are shown in Table 1. ESS had the highest coefficient of variation of 21.4%. High positive phenotypic and genetic correlations among them are presented in Table 2.

**Table 1:** Descriptive statistical analysis of eggshell mechanical property.

Traits^a^	Number	Average	S.D.	Min	Max	CV (%)
**ESS (kg/cm** ^**2**^ **)**	973	2.644	0.566	0.929	5.499	21.4
**EST (mm)**	976	0.32	0.023	0.224	0.39	7.13
**ESW (g)**	976	5.9	0.5	3.9	7.1	8.13

^a^ESS = Eggshell Strength; EST = Eggshell Thickness; ESW = Eggshell Weight.

**Table 2:** Phenotypic and genetic correlation among eggshell mechanical property and heritability^a^.

Traits^b^	ESS (kg/cm^2^)	EST (mm)	ESW (g)
**ESS (kg/cm** ^**2**^ **)**	-	0.5597	0.4212
**EST (mm)**	0.5893(0.1156)	-	0.7487
**ESW (g)**	0.2988(0.1492)	0.7357(0.0716)	-

^a^Phenotypic correlations are given above diagonal, and genetic correlations below diagonal. Standard errors of estimates are in parentheses.

^b^ESS = Eggshell Strength; EST = Eggshell Thickness; ESW = Eggshell Weight.

### SNP Summary and Haplotype Construction

The distribution of 99 SNPs in 15 genes and the information of gene location are presented in S2 Table. Genotype quality control and data filtering resulted in the removal of 24 SNPs with a low minor allele frequency (<1%) and 54 SNPs with monopolymorphism. The remaining twenty-one SNPs in nine genes that agreed with Hardy-Weinberg equilibrium were finally identified as polymorphic with MAF >1% and genotype call rates >90% (S3 Table).

To further detect multi-loci association of each gene with eggshell mechanical property traits, linkage disequilibrium (LD) among SNPs within each gene were analyzed using the Haploview program, with the default algorithm and default parameter settings, and SNPs that deviated from the Hard-Weinberg equilibrium were removed. Strong LD was detected within each of the four ion transport genes, i.e. *ATP2A3*, *ITPR1*, *SLC8A3* and *SCNN1a*, and each of them had one LD block (S1 Fig). Haplotypes were constructed for SNPs within the same LD block. The major haplotypes (frequency >1%) and diplotypes (frequency >1%) in four LD blocks were shown in Table 3.

**Table 3:** Major haplotypes (frequency >1%) and diplotypes (frequency >1%) in 4 linkage disequilibrium (LD) blocks.

LD block	Haplotypes (%)	Diplotypes (%)
**LD-ATP2A3**	H1: CC (14.27), H2: TT (85.67)	H1H1 (1.64), H1H2 (25.20), H2H2 (73.05)
**LD-ITPR1**	H1: GGCCGGTC (23.97), H2: GGTTGACT (33.64), H3: AACCAGTC (41.44)	H1H1 (4.92), H1H2 (14.55), H1H3 (22.95), H2H2 (11.99), H2H3 (28.59), H3H3 (15.27)
**LD-SLC8A3**	H1: CT (51.75), H2: TC (48.15)	H1H1 (27.08), H1H2 (49.84), H2H2 (23.08)
**LD-SCNN1a**	H1: ATT (57.06), H2: GTT (22.28), H3: GCC(20.55)	H1H1 (33.50), H1H2 (23.97), H1H3 (23.87), H2H2 (4.71), H2H3 (10.35), H3H3 (3.38)

### Association of Single SNP with Eggshell Mechanical Property Traits

The results showed that 14 SNPs in 8 genes were significantly associated (*p* < 0.05) with at least one trait, and the CPV of these SNPs were presented in Table 4. ESS had significant association with three SNPs, rs14118603 in *ATP2A3*, rs14075350 in *SCNN1b* and ss538155652 in *SLC4A5* (p <0.05). The CPV of these three SNPs to ESS ranged from 0.46% to 1.11%. For EST, nine SNPs were significantly associated (*p* < 0.05), and they were located in four genes: *ATP2A3*, *ITPR1*, *KCNMA1* and *SLC4A5*, and the CPV of these SNPs to EST ranged from 0.33% to 1.30%. And for ESW, six SNPs showed significant association (*p* < 0.05), and they were located in six genes: *ATP2A3*, *CA7*, *KCNMA1*, *SCNN1a*, *SCNN1g* and *SLC4A5*. The CPV of these SNPs ranged from 0.23% to 4.14%. Especially, the SNP ss538155652 in *SLC4A5* exhibited significant association with all the traits, and had a high CPV to EST (1.30%).

**Table 4:** Association analysis of the SNPs with eggshell mechanical property traits^a^ ^,^ ^b^ ^,^ ^c^.

SNP	Gene	Region	ESS	EST	ESW
**rs15841856**	ATP2A3	ex7	0.2395	0.0386* (0.5290)	0.1252
**rs13574212**	ATP2A3	ex20	0.2791	0.0452* (0.4834)	0.1325
**rs14118603**	ATP2A3	ex22	0.024* (0.6837)	0.0707	<.0001** (4.141)
**rs14964612**	CA7	5'UTR	0.8680	0.4916	0.0439* (0.2344)
**rs14986134**	ITPR1	ex4	0.3056	0.0136* (0.5227)	0.6505
**rs15672233**	ITPR1	ex44	0.2647	0.0052** (0.6892)	0.4181
**rs15672283**	ITPR1	ex51	0.2672	0.0056** (0.6812)	0.4576
**rs15672301**	ITPR1	ex54	0.2090	0.0068** (0.6650)	0.5083
**rs15672305**	ITPR1	ex54	0.2581	0.005** (0.7092)	0.4742
**rs16544657**	KCNMA1	3'UTR	0.3436	0.0325* (0.3266)	0.0001** (1.251)
**rs13886291**	SCNN1^a^	ex3	0.3454	0.3770	0.0074** (0.6122)
**rs14075350**	SCNN1^b^	5'UTR	0.0043** (1.109)	0.3304	0.7113
**rs15009190**	SCNN1g	3'UTR	0.9615	0.5218	0.0451* (0.2354)
**ss538155652**	SLC4A5	3'UTR	0.0366* (0.4568)	0.0011** (1.302)	0.0045** (0.7858)

^a^ESS = Eggshell Strength; EST = Eggshell Thickness; ESW = Eggshell Weight.

^b^* *p* < 0.05;

** *p* < 0.01.

^c^SNP contribution to phenotypic variance (%) are in parentheses.

### Association of Haplotypes with Eggshell Mechanical Property Traits

Haplotypes had combined effect of SNPs on phenotypes, and their association analysis with eggshell mechanical property traits was performed. The CPV of these diplotypes to eggshell mechanical traits are presented in Table 5. The results showed that there was significant association (p <0.05) between diplotypes and eggshell mechanical property traits, except *SLC8A3*. Effects of the diplotypes in each LD block on eggshell mechanical property traits were listed in S4 Table.

**Table 5:** Association analysis of the diplotypes with eggshell mechanical property traits^a^ ^,^ ^b^ ^,^ ^c^.

LD-block	Gene	ESS	EST	ESW
**LD- ATP2A3**	ATP2A3	0.2834	0.0427*(0.50)	0.1345
**LD- ITPR1**	ITPR1	0.4261	0.0030**(0.70)	0.5788
**LD- SCNN1a**	SCNN1^a^	0.4918	0.3281	0.0122* (0.54)
**LD- SLC8A3**	SLC8A3	0.5800	0.2276	0.4723

^a^ESS = Eggshell Strength; EST = Eggshell Thickness; ESW = Eggshell Weight.

^b^* *p* < 0.05;

** *p* < 0.01.

^c^Diplotype contribution to phenotypic variance (%) are in parentheses

No significant association was found between ESS and diplotypes within *ATP2A3*, *ITPR1*, *SCNN1a* and *SLC8A3*. The diplotypes within *ATP2A3* and *ITPR1* were significantly (*p* <0.05) associated with EST, and they all had low CPVs (< 0.70%). The diplotypes within *SCNN1a* were significantly (*p* <0.05) associated with ESW, but it had a low CPV (0.54%).

## Discussion

Eggshell breaking strength, thickness and weight reflect mechanical properties of eggshell in different aspects and are the most common parameters for measuring the ability of resisting exterior force [26–29]. Additionally, they can indicate the final state of eggshell mineralization after a complex ions transport.

The identification of ion transporters related to eggshell mineralization could improve our understanding of the mechanisms and regulation for ionic precursors of calcium carbonate (CaCO_3_), and enable us to find new potential genes effectively. In this study, 15 ion transport genes were analyzed for their genetic effect on eggshell mechanical properties for the first time. The association analysis results showed that 78 SNPs were filtered regrettably for their low minor allele frequencies (<1%) or monopolymorphism in the current Rhode Island Red population. This strain has been chosen for 9 generations in accordance with the appearance, growth and egg weight traits within every generation. At last, 14 SNPs from 8 genes were found significantly associated with eggshell mechanical traits. These 8 genes involved four kinds of ions, Ca^2+^, HCO_3_^-^, Na^+^ and K^+^. This indicated that the eggshell mineralization was a collaboration process of multiple ions transport and regulation, which was supported by the differential expression of genes involved in those ions between uteruses in mineralization and non-mineralization in a microarray study [30].

The transport of Ca^2+^ and HCO_3_^-^ is the core of ion transport in uterus, which has a great effect on eggshell mineralization and eggshell quality. The amount of calcium deposited in eggshell is about 2 g, almost 10% of the total body calcium [17, 31]. However, both of carbonate and calcium ions are not stored in the uterus before eggshell mineralization but are continuously transported from the blood plasma through uterine endothelium [7, 31]. Thus, laying birds have severe demands on calcium homeostasis and more active ions transport than non-laying animals [16, 32–33]. In this study, the Ca^2+^ transport genes *ATP2A3* and *ITPR1*, and the HCO_3_^-^ transport genes *CA7* and *SLC4A5* had significant genetic effects on eggshell mechanical properties. *ATP2A3* were expressed in many types of tissues, such as platelets, lymphoid cells and mast cells in human [34], which were not only involved in calcium transport but also relevant to protein folding in endoplasmic reticulum (ER) [35–37]. In uterine glandular cells, *ATP2A3*, localised in the endoplasmic reticulum, played a role in calcium dynamic store and maintaining the low level of free Ca^2+^ in cytoplasm [2], which may relate to the supply of calcium for eggshell formation, and be more likely relevant to eggshell matrix protein folding. In the present study, three SNPs in *ATP2A3* were found significant association with EST, ESS and ESW. Moreover, these three SNPs were located in the same linkage disequilibrium block, and the corresponding haplotypes were significantly associated with EST. *ITPR1* was another gene which was directly involved in Ca^2+^ transport, and mainly localised in the endoplasmic reticulum, releasing Ca^2+^ from the endoplasmic reticulum [38–39]. The expression of *ITPR1* in uterus was higher than in magnum and duodenum, and there was no difference between the different processes of presence and absence of eggshell formation in uterus [2]. In this study, five SNPs in *ITPR1* were significantly associated with EST. These five SNPs together with rs15672050, rs15672053 and rs14986199 fall in the same LD block significantly associated with EST.

*CA7* and *SLC4A5* are genes transporting HCO_3_^-^. *CA7* belongs to a family of enzymes which can catalyze carbon dioxide and water to bicarbonate and protons, and functions in cytoplasm [40]. The expression study showed that *CA7* had a higher expression in chicken uterus than in magnum [2]. Previous studies suggested that most HCO_3_^-^ for eggshell calcification came from the blood CO_2_, rather than the plasma HCO_3_^-^ [15, 41]. In the current study, rs14964612 in *CA7* was significantly associated with ESW, indicating that *CA7* played an important role in supply of HCO_3_^-^. *SLC4A5* is a Na^+^/ HCO_3_^-^ cotransporter, as predicted in the basolateral membrane of uterine glandular cells to allow the entry of HCO_3_^-^. It was shown higher expression in uterus than in magnum and duodenum [2]. Present results showed that ss538155652 in *SLC4A5* was significantly associated with EST, which provided evidence for the great effect of *SLC4A5* on the transport of HCO_3_^-^.

*SCNN1a*, *SCNN1b* and *SCNN1g* belong to the sodium channel gene family, which encode three subunits of epithelial Na^+^ channel respectively [42]. These three subunits showed higher expression in uterus in chicken compared to magnum, duodenum, liver, and kidney [2, 29], indicating their importance in uterine Na^+^ transport. We found that rs13886291 (*SCNN1a*) and rs15009190 (*SCNN1g*) had a significant effect on ESW, and rs14075350 in *SCNN1b* on ESS. The haplotype block of LD-*SCNN1a* (rs13886291, rs14845041) was significantly associated with ESW.

K^+^ channels play a crucial role for many complex biological processes, including cell volume regulation, cell migration, differentiation and apoptosis [43]. Variety of K^+^ channels provide energy for many voltage-driven transport processes, such as bicarbonate secretion in small intestinal villus cells, electrogenic glucose reabsorption in small intestine, and colonic Na^+^ reabsorption by epithelial Na^+^ channels [43–44]. We found that rs16544657 in *KCNMA1* (K^+^ large conductance Ca^2+^ activated channels, subfamily M) had a significant association with EST and ESW. In chicken, the higher expression of *KCNMA1* in active uterus and the increased K^+^ concentrations in uterine fluid between early and late stages of eggshell calcification indicated the active and important role of K^+^ channels in uterus physiological function, or uterine mineralization [2].

## Conclusion

This study evaluated the association between uterine ion transport genes and eggshell mechanical property traits for the first time. The association analysis provided evidences that 14 SNPs in 8 genes were significantly associated (*p* < 0.05) with at least one trait. Both *ATP2A3* and *SLC4A5* had a significant effect on all the traits. The rs14118603 in *ATP2A3* could contribute 4.14% to the variation of eggshell weight. The results supported that eggshell mineralization was a collaboration process of multiple ions transport and regulation. Identified SNPs and haplotypes in this study will help understand the process of ion transport during eggshell formation, and these potential markers may be available to the genetic improvement in eggshell quality.

## Supporting Information

S1 Fig Linkage disequilibrium analysis for SNPs in 6 ion transport genes respectively. (TIF)

S1 Table The 99 pairs of primers used in this study. (XLSX)

S2 Table The location of the ion transport genes and the distribution of selected SNPs in each of them. ^a^No. of SNP = the number of the SNP selected in each gene
(DOCX)

S3 Table The detail information of SNPs and their MAF and call rates in the experiment population. ^a^MAF = Minor Allele Frequency.
(DOCX)

S4 Table Effect of the diplotypes on eggshell mechanical property traits. ^**a**^LSM = least squares means. The values with different letters means significant difference (p value < 0.05); ^b^SE = standard error.
(XLSX)
