# Supplementary figures and images for: Polymorphisms in Ion Transport Genes Are Associated with Eggshell Mechanical Property
Source: PLoS One. 2015 Jun 24;10(6):e0130160. doi: 10.1371/journal.pone.0130160 (PMC4481273; doi:10.1371/journal.pone.0130160)

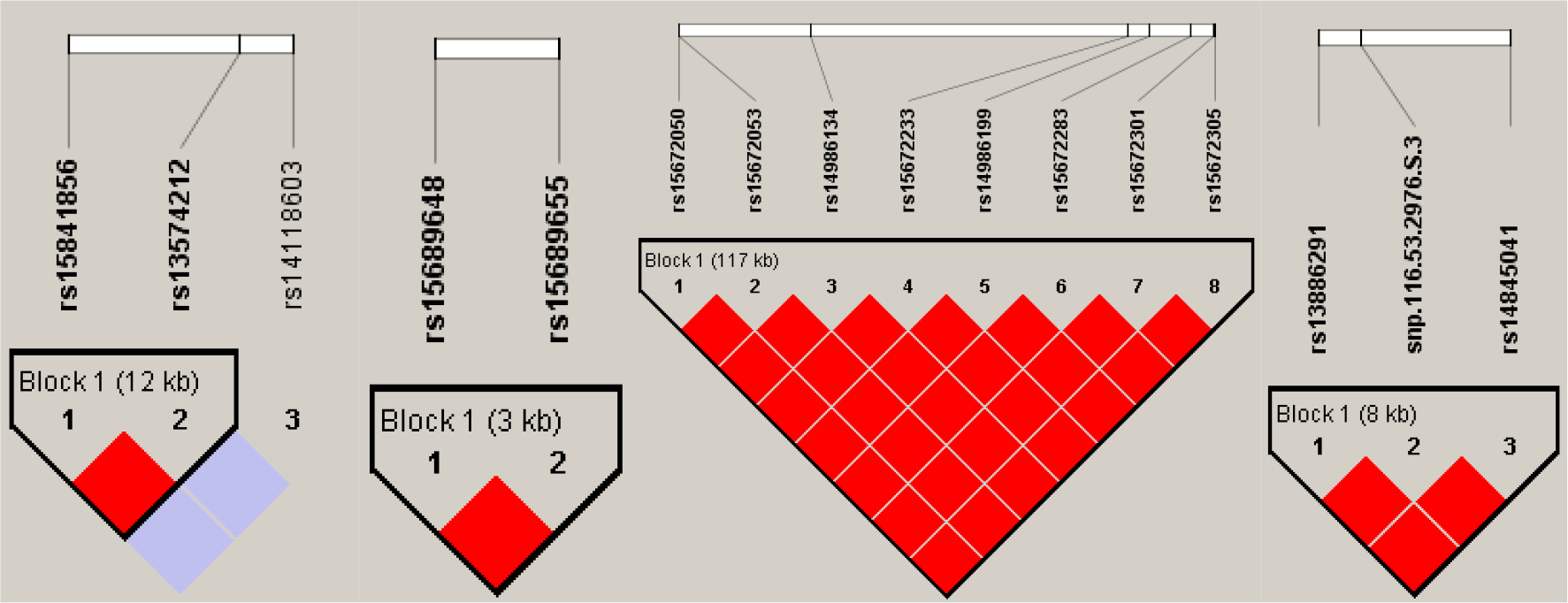

Supplement: S1 Fig — (TIF) [file pone.0130160.s001.tif]
