# Supplementary material for: Polymorphisms in Ion Transport Genes Are Associated with Eggshell Mechanical Property
Source: PLoS One. 2015 Jun 24;10(6):e0130160. doi: 10.1371/journal.pone.0130160 (PMC4481273; doi:10.1371/journal.pone.0130160)
Supplement: S2 Table — aNo. of SNP = the number of the SNP selected in each gene (DOCX) [file pone.0130160.s003.docx]

**S2 Table. The location of the ion transport genes and the distribution of selected SNPs in each of them**

| **Gene** | **Gene location** | **No. of SNP^a^** |
| --- | --- | --- |
| **ATP2A3** | Chr19: 3,239,944-3,264,151 | 10 |
| **ATP2B1** | Chr1: 45,276,215-45,305,806 | 7 |
| **ATP2B2** | Chr12: 4,060,351-4,223,100 | 4 |
| **CA7** | Chr11: 12,174,230-12,183,227 | 3 |
| **CLCN5** | Chr4: 9,635,234-9,660,403 | 7 |
| **ITPR1** | Chr12: 19,042,283-19,181,612 | 15 |
| **ITPR2** | Chr1: 69,818,939-70,056,320 | 9 |
| **KCNJ16** | Chr18: 8,451,086-8,452,375 | 6 |
| **KCNJ2** | Chr18: 8,205,149-8,211,893 | 6 |
| **KCNMA1** | Chr6: 14,820,491-14,858,126 | 3 |
| **SCNN1a** | Chr1: 80,034,908-80,045,394 | 5 |
| **SCNN1b** | Chr14: 7,002,221-7,011,192 | 7 |
| **SCNN1g** | Chr14: 7,019,365-7,028,441 | 4 |
| **SLC4A5** | Chr22_random: 17,335-47,303 | 4 |
| **SLC8A3** | Chr5: 29,914,505-29,994,536 | 9 |

^a^No. of SNP = the number of the SNP selected in each gene.
