# Supplementary material for: Polymorphisms in Ion Transport Genes Are Associated with Eggshell Mechanical Property
Source: PLoS One. 2015 Jun 24;10(6):e0130160. doi: 10.1371/journal.pone.0130160 (PMC4481273; doi:10.1371/journal.pone.0130160)
Supplement: S3 Table — aMAF = Minor Allele Frequency. (DOCX) [file pone.0130160.s004.docx]

**S3 Table. The detail information of SNPs and their MAF and call rates in the experiment population**

| **Gene** | **SNP** | **Chromosome** | **Position** | **Alleles** | **Region** | **MAF(%)** | **Call (%)** | **p value** |
| --- | --- | --- | --- | --- | --- | --- | --- | --- |
| **ATP2A3** | rs15841856 | 19 | 3246973 | C/T | Exon7 | 14.28 | 98.67 | 0.4287 |
| **ATP2A3** | rs13574212 | 19 | 3259674 | C/T | Exon20 | 14.27 | 99.80 | 0.3607 |
| **ATP2A3** | rs14118603 | 19 | 3263565 | A/G | Exon22 | 26.35 | 98.77 | 0.9339 |
| **CA7** | rs14964612 | 11 | 12183783 | C/G | 5'UTR | 31.73 | 99.28 | 0.8242 |
| **ITPR1** | rs15672050 | 12 | 19041571 | G/A | 5'UTR | 42.06 | 96.11 | 0.0520 |
| **ITPR1** | rs15672053 | 12 | 19041748 | G/A | 5'UTR | 41.69 | 98.05 | 0.0115 |
| **ITPR1** | rs14986134 | 12 | 19070575 | C/T | Exon4 | 33.97 | 97.13 | 0.4259 |
| **ITPR1** | rs15672233 | 12 | 19140349 | C/T | Exon44 | 34.02 | 97.44 | 0.4706 |
| **ITPR1** | rs14986199 | 12 | 19145142 | A/G | Exon45 | 41.74 | 97.34 | 0.0327 |
| **ITPR1** | rs15672283 | 12 | 19154050 | A/G | Exon51 | 33.95 | 97.64 | 0.5633 |
| **ITPR1** | rs15672301 | 12 | 19159254 | C/T | Exon54 | 34.24 | 98.46 | 0.5200 |
| **ITPR1** | rs15672305 | 12 | 19159266 | T/C | Exon54 | 34.45 | 97.85 | 0.4306 |
| **KCNMA1** | rs16544657 | 6 | 14863096 | C/T | 3'UTR | 47.74 | 97.34 | 0.2694 |
| **SCNN1A** | rs13886291 | 1 | 80036082 | G/A | Exon3 | 42.74 | 97.44 | 0.6910 |
| **SCNN1A** | snp.116.53.2976.S.3 | 1 | 80038004 | C/T | Exon5 | 20.56 | 99.18 | 0.1396 |
| **SCNN1A** | rs14845041 | 1 | 80044519 | T/C | Exon13 | 20.53 | 99.08 | 0.0939 |
| **SCNN1B** | rs14075350 | 14 | 7011699 | C/T | 5'UTR | 42.83 | 97.95 | 0.0644 |
| **SCNN1G** | rs15009190 | 14 | 7017446 | T/C | 3'UTR | 30.95 | 95.49 | 0.8182 |
| **SLC4A5** | ss538155652 | 22 | 15469 | G/T | 3'UTR | 42.53 | 97.34 | 0.3885 |
| **SLC8A3** | rs15689648 | 5 | 29912507 | T/C | 5'UTR | 48.05 | 97.34 | 1.0000 |
| **SLC8A3** | rs15689655 | 5 | 29915815 | C/T | Exon1 | 48.03 | 98.98 | 0.9486 |

^a^MAF = Minor Allele Frequency
